# Metabolomics analysis of SNAT2-deficient cells: Implications for the discovery of selective small-molecule inhibitors of an amino acid transporter

**DOI:** 10.1016/j.jbc.2025.110525

**Published:** 2025-07-25

**Authors:** Jessica C. Koe, Yuxi C. Zhong, Kiana Pashaoskooie, Gregory J. Kaczorowski, Maria L. Garcia, Seth J. Parker

**Affiliations:** 1 Department of Biochemistry & Molecular Biology, University of British Columbia, Vancouver, British Columbia, Canada; 2 Kanalis Consulting, LLC, Edison, New Jersey, USA; 3 Centre for Molecular Medicine and Therapeutics, University of British Columbia, Vancouver, British Columbia, Canada; 4 British Columbia Children’s Hospital Research Institute, Vancouver, British Columbia, Canada

**Keywords:** amino acid transporters, solute carriers, metabolism, SLC38A2, SNAT2, mass spectrometry, metabolomics

## Abstract

Amino acid uptake by the solute carrier family of transporter proteins is critical to support cell metabolism, and inhibition of transporter activity represents a tractable strategy to restrict nutrient availability to cancer cells. A small-molecule inhibitor of the sodium-coupled neutral amino acid transporter 2 (SNAT2), 3-(*N*-methyl(4-methylphenyl)sulfonamido)-*N*-(2-trifluoromethylbenzyl)thiophene-2-carboxamide (MMTC/57E), was recently identified and shown to inhibit cell proliferation when combined with glucose transport inhibitors in breast and pancreatic cancer cell lines. In this study, we use mass spectrometry and a model competitive substrate inhibitor, α-(methylamino)-isobutyric acid (MeAIB), to establish cell-based SNAT2 activity assays and validate target engagement of MMTC/57E. We show that cellular uptake of MeAIB is dependent on SNAT2 or the closely related SNAT1 and is inhibited by the endogenous substrate l-alanine in a dose-dependent manner. We show that SNAT2-KO cells or cells treated with MeAIB exhibit a similar metabolomic signature associated with defects in amino acid availability and other metabolites. Applying these assays, we fail to observe that MMTC/57E inhibits SNAT2 activity. MMTC/57E exhibits poor aqueous solubility that hinders its use as a tool SNAT2 inhibitor. Our results highlight the challenges associated with identifying and validating transporter inhibitors and report robust assays that may be used to identify and evaluate SNAT2 inhibitors in the future.

The sodium-coupled neutral amino acid transporter 2 (SNAT2/SLC38A2) functions as a sodium-dependent cotransporter of small neutral amino acids, such as alanine, asparagine, glutamine, methionine, serine, and threonine (1, 2). Substrates of SNAT2 fuel protein synthesis, cellular bioenergetics, amino acid metabolism, and *de novo* lipogenesis, and as such, SNAT2 has been suggested as a potential therapeutic target for human cancers. In breast cancer cell lines, SNAT2 deficiency has been shown to reduce glutamine consumption and cell growth under hypoxia, indicating the importance of SNAT2 in sensitizing cells to oxidative stress (3, 4). Furthermore, knocking out SNAT2 expression leads to impaired alanine uptake and reduced proliferation of human and mouse pancreatic ductal adenocarcinoma (PDAC) cell lines and was sufficient to cause tumor regression in mouse tumor models (5). The identification of potent and selective SNAT2 inhibitors is needed to explore the efficacy of SNAT2 as a therapeutic target for cancer.

Given the importance of SNAT2 for amino acid transport and tumor metabolism, we were interested in a recent report by Gauthier-Coles *et al.* (6), which reported the identification of several high-affinity SNAT2 inhibitors, including 3-(*N*-methyl(4-methylphenyl)sulfonamido)-*N*-(2-trifluoromethylbenzyl)thiophene-2-carboxamide (MMTC, also referred to as “57E”). The authors established an FLIPR membrane potential assay to measure glutamine-dependent membrane depolarization in amino acid–starved HCC1806 breast cancer cells (6). SNAT1 and SNAT2 are robustly expressed on the cell surface of these cells under these conditions and were suggested to contribute to the glutamine-dependent depolarization signal, which was inhibited by the glutamine analog γ-glutamylhydroxamate (6). Using this assay, the authors conducted a high-throughput screen using a curated library of ∼34,000 compounds and identified several compounds with low micromolar IC_50_ values as inhibitors of glutamine uptake, including the lead compound MMTC/57E (6). However, a follow-up study by Jakobsen *et al.* (7) indicated that 57E—as well as other hit compounds 53B, 54F, 55B, and 57B identified by Gauthier-Coles *et al.*—failed to inhibit SNAT2-dependent ^3^H-glycine uptake in hyperosmotic-stressed PC3 cells.

Given the discrepancies concerning the activity of the aforementioned compounds and toward identifying potent and selective SNAT2 inhibitors, we developed a mass spectrometry (MS)–based readout of SNAT2 activity and cell lines suitable to determine inhibitor target engagement. Using metabolomics, we show that SNAT2-deficient cells fail to sequester most proteinogenic amino acids and display a similar metabolic signature as cells treated with the well-validated tool inhibitor of SNAT2, α-(methylamino)-isobutyric acid (MeAIB) (8, 9). In agreement with Jakobsen *et al.*, we failed to observe evidence that MMTC/57E inhibits SNAT2 activity up to a concentration of 40 μM. Using LC–MS, we showed that the MMTC/57E exhibits poor aqueous solubility and was completely removed after centrifugation and filtration. Our results highlight the challenges in identifying specific and selective amino acid transporter inhibitors, stressing the need for multiple orthogonal assays for compound screening and selectivity assessment.

## Results

### Developing a cell line to dissect system a transporter activity

We have previously shown that SNAT2 is highly expressed and constitutively active in several human PDAC cell lines, human PDAC tumors, and cell lines established from primary mouse “KPC” (*LSL-KrasG12D; Trp53lox/+; p48Cre+*) tumors (5). Amino acid transporter expression profiles in any given cell line, especially those that share substrates with SNAT2, are expected to influence how individual transporters contribute to transport systems and coordinate substrates to fuel cell metabolism. Furthermore, it is well established that intracell line heterogeneity and selective pressures during the cloning process can influence clonal phenotypes when comparing nonisogenic clones (10). For these reasons, we had previously established an isogenic clonal cell line isolated from the HY15549 mouse KPC cell line that harbors a CRISPR–Cas9-mediated deletion of endogenous *Slc38a2* and expresses a doxycycline (dox)-inducible single guide RNA–resistant human *SLC38A2* complementary DNA in tandem, herein referred to as “HY15549 iSNAT2” (5). To limit selective pressures associated with Snat2 deficiency during clonal generation, dox-induced SNAT2 expression was maintained throughout subcloning and clone validation (5). We rationalized that this isogenic cell system represents a suitable cell line to allow us to understand the metabolic and cellular effects caused by acute SNAT2 deficiency and to develop SNAT2 activity assays to assess target inhibition.

**Figure 1:**
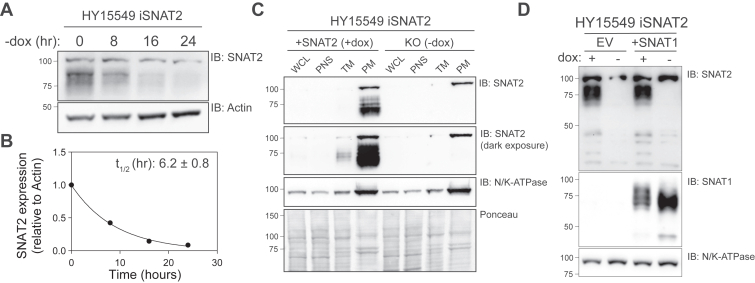
**Development of a cell line to characterize SNAT2 activity.***A* and *B*, SNAT2 immunoblot in HY15549 iSNAT2 after withdrawal of doxycycline (dox) over the course of 24 h. Densitometry analysis, relative to a parallel actin immunoblot, suggests an SNAT2 half-life of approximately 6 h after dox withdrawal. *C*, SNAT2 and sodium/potassium (N/K)-ATPase immunoblots in whole cell lysate (WCL), postnuclear supernatant (PNS), total membrane (TM), and plasma membrane (PM) samples isolated from HY15549 iSNAT2 cells cultured with or without dox (0.5 μg/ml) for 24 h. An equal amount of protein (20 μg) was loaded for each sample, which was confirmed using a Ponceau S stain. *D*, SNAT1, SNAT2, and N/K-ATPase immunoblots in HY15549 iSNAT2 EV or + SNAT1 cell lines cultured with or without dox for 24 h. Each immunoblot is representative of n ≥ 1 independent experiments. EV, empty vector; SNAT2, sodium-coupled neutral amino acid transporter 2.

We first analyzed the turnover of SNAT2 following dox withdrawal using Western blotting. The expression of mature, glycosylated SNAT2 rapidly extinguished with a half-life of ∼6 h with minimal protein detected after 24 h (Fig. 1, *A* and *B*). To confirm that ectopically expressed SNAT2 is localized to the cell surface, we performed a total membrane capture and subsequent enrichment of plasma membrane fragments by sucrose density gradient ultracentrifugation (11). This method robustly enriched glycosylated SNAT2 in cells maintained with 0.5 μg/ml dox (Fig. 1*C*). Withdrawal of dox for ∼24 h led to a virtually complete depletion of plasma membrane–localized SNAT2 (Fig. 1*C*). Next, we analyzed the expression of the system A transporter SNAT1 in HY15549 iSNAT2 cells to verify the possibility of confounding effects by homologous transporters in downstream assays. SNAT1 and SNAT2 are closely related, are highly homologous, have similar substrate profiles and transport mechanisms, and are both competitively inhibited by MeAIB (8, 9). HY15549 iSNAT2 cells were stably transduced with a cytomegalovirus (CMV)-driven human *SLC38A1* complementary DNA (+SNAT1) or an empty vector (EV) control and analyzed by Western blot. We observed low, undetectable expression of Snat1 in HY15549 iSNAT2 and confirmed overexpression of SNAT1 in cells constitutively overexpressing human *SLC38A1* (Fig. 1*D*).

### Establishing an assay to characterize SNAT2-specific activity

**Figure 2:**
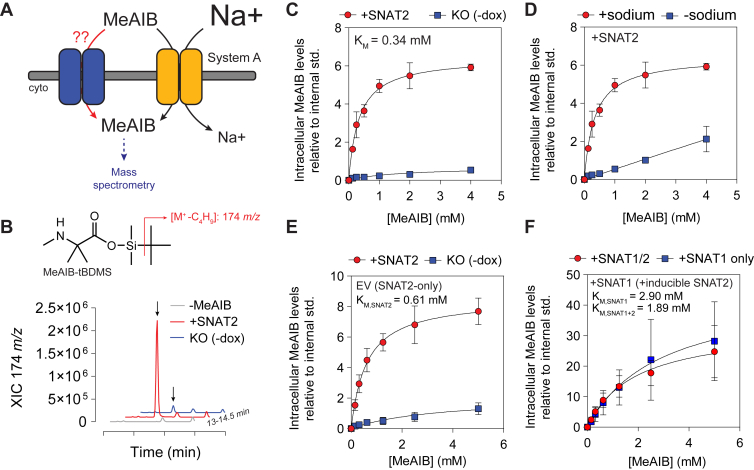
**MeAIB uptake using GC–MS quantifies SNAT1 or SNAT2 activity.***A*, schematic showing the sodium-dependent uptake of MeAIB by system A transporters, as quantified by GC–MS. *B*, extracted ion chromatogram (XIC) of representative GC–MS samples for 174 *m/z*, corresponding to the main fragment ion of tertbutyldimethylsilylated MeAIB. *C*, MeAIB uptake in HY15549 iSNAT2 cells cultured with (+SNAT2) or without (SNAT2-KO) doxycycline (dox). *D*, MeAIB uptake in HY15549 iSNAT2 cells cultured with dox performed in PBS with or without sodium. *E* and *F*, MeAIB uptake in HY15549 iSNAT2 EV (*E*) or +SNAT1 (*F*) cells cultured with or without dox. *C*–*F*, all MeAIB uptake assays were performed 24 h after plating with or without dox to allow for SNAT2 expression to extinguish, in the case of no supplemented dox. Data in (*C*–*F*) are mean ± SD of n = 3 independent experiments; the Michaelis–Menten constant (*K*_*M*_) was estimated using a nonlinear fit in Prism. MeAIB, α-(methylamino)-isobutyric acid; SNAT1/2, sodium-coupled neutral amino acid transporter 1/2.

Cell-based, functional assays are among the most robust and tractable assay types amenable for characterizing inhibitor target engagement. We sought to identify a substrate that would be transported into cells in a highly SNAT2-specific manner and that could be measured using widely available instrumentation. Radiolabeled [^14^C]MeAIB transport studies in BeWo choriocarcinoma cells suggest that MeAIB is a transepithelial transported substrate (12), but radiolabeled substrates are expensive and complex to handle and dispose. Therefore, we sought to establish a transport assay to measure SNAT2-dependent uptake of non–radioisotope-labeled MeAIB using GC-MS (Fig. 2*A*). MeAIB is readily derivatized *via* tertbutyldimethylsilylation (tert-butyldimethylsily [TBDMS]), which yields a derivative with a main fragment ion of 174 *m/z* corresponding to [M-57] (-C4H9, *tert*-butyl) (Fig. 2*B*). This feature was not seen in nontreated, control samples (Fig. 2*B*). Integration of this peak revealed saturable MeAIB uptake with an approximate Michaelis–Menten constant (*K*_*M*_) of ∼0.3 mM, which was dependent on SNAT2 expression (Fig. 2*C*). SNAT2-KO led to nearly negligible MeAIB uptake, further supporting the idea that SNAT2 is the primary system A transporter in this cell line (Fig. 2*C*). When cells expressing SNAT2 (+dox) were incubated in a sodium-free transport buffer in which sodium chloride was substituted with equimolar *N*-methyl-d-glucamine and other sodium salts were replaced with potassium salts, MeAIB uptake was significantly attenuated consistent with the sodium dependence of SNAT2 (Fig. 2*D*). Taken together, MS-based measurements of MeAIB uptake provide a specific and sensitive readout of SNAT2 activity.

As SNAT1 and SNAT2 represent potentially redundant neutral amino acid transporters, we aimed to determine whether SNAT1 could also facilitate MeAIB uptake. For this purpose, we quantified MeAIB uptake kinetics using GC–MS in HY15549 SNAT2-KO expressing human SNAT1 or EV controls. EV cells expressing exclusively SNAT2 exhibited similar MeAIB uptake kinetics (*K*_*M*_ of 0.6 mM) as the nontransduced HY15549 iSNAT2 cells cultured with or without dox (Fig. 2, *C* and *E*). Cells overexpressing SNAT1 also exhibited MeAIB uptake, independent of SNAT2 expression status, with a *K*_*M*_ of ∼2.9 mM, which is ∼5–10× lower than the affinity of MeAIB for SNAT2 (Fig. 2*F*). Notably, the reported *K*_*M*_ of structurally similar l-alanine for rat SNAT1 and SNAT2 is 0.35 mM and 0.2 mM, respectively (13, 14, 15). SNAT1 and SNAT2 share broad overlapping preference for other small neutral amino acids (1, 2). It is possible that SNAT1 and SNAT2 may exhibit significantly different affinities for other substrates, which has been previously explored and suggested to contribute to nutrient homeostasis and sensing in limiting environments (16). Although the magnitude of MeAIB uptake also significantly differed between SNAT1- or SNAT2-expressing cells, this is likely because of a difference in the absolute level of overexpression and/or surface expression. Taken together, MeAIB uptake measured using MS can be used to assay the relative activity of SNAT1 and/or SNAT2.

### MMTC/57E fails to inhibit SNAT2-dependent MeAIB uptake

**Figure 3:**
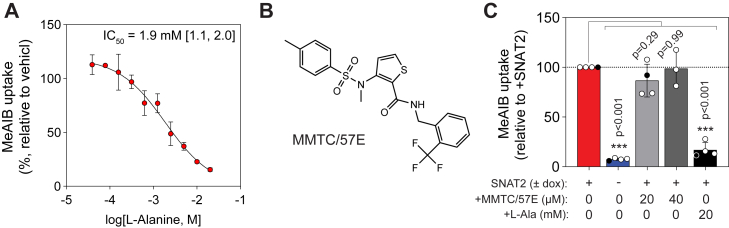
**MMTC/57E does not significantly inhibit SNAT2-dependent MeAIB uptake.***A*, concentration response curve to determine the IC_50_ for l-alanine inhibition of MeAIB uptake in HY15549 iSNAT2 cells. Cells were cultured with doxycycline (dox) and coincubated with 0 to 20 mM of l-alanine and 0.5 mM of MeAIB for 30 min. *B*, structure of MMTC/57E. *C*, MeAIB uptake in HY15549 iSNAT2 cells cultured without dox for 24 h (“KO”), or cells cultured with dox (+SNAT2) treated with 20 or 40 μM of MMTC/57E or 20 mM of l-alanine; all data are relative to vehicle-treated (DMSO) + SNAT2 cells. *Black circles* represent an independent experiment whereby a predilution of the MMTC/57E was performed in DMSO to limit solubility issues. Data are mean ± SD of n ≥ 3 independent experiments; the IC_50_ value for l-alanine was estimated using a nonlinear fit in Prism, and the 95% confidence interval is listed in *brackets*. Significance was determined by a one-way ANOVA using Dunnett’s multiple comparisons test and a family wise alpha threshold of 0.05. DMSO, dimethyl sulfoxide; MeAIB, α-(methylamino)-isobutyric acid; MMTC/57E, 3-(*N*-methyl(4-methylphenyl)sulfonamido)-*N*-(2-trifluoromethylbenzyl)thiophene-2-carboxamide; SNAT2, sodium-coupled neutral amino acid transporter 2.

To validate that the SNAT2-dependent cellular uptake of MeAIB is sensitive to competition by known substrates, we quantified the uptake of 0.5 mM MeAIB in cells cotreated with a concentration gradient of l-alanine between 0 and 20 mM. l-alanine inhibited MeAIB uptake with an IC_50_ of 1.9 mM (Fig. 3*A*). We next acquired the reported SNAT2 inhibitor MMTC/57E to validate its capacity to inhibit MeAIB uptake (Fig. 3*B*). The previously reported IC_50_ for MMTC/57E was 0.8 μM, with near maximal inhibition of [^14^C]proline uptake at concentrations >10 μM^6^. We treated SNAT2 cells with either 20 or 40 μM MMTC/57E and quantified the effects on MeAIB uptake relative to either SNAT2-KO (24-h dox withdrawal, as before) or cells treated with l-alanine at ∼10× its IC_50_ (20 mM). For these experiments, cells were pretreated with either MMTC/57E or l-alanine for 15 min before the addition of 0.5 mM MeAIB for 30 min at room temperature. As expected, SNAT2-KO and l-alanine treatment significantly suppressed MeAIB uptake by 92% and 83%, respectively (Fig. 3*C*). However, we failed to observe a significant reduction in SNAT2-dependent MeAIB uptake with either 20 or 40 μM of MMTC/57E across 3 independent experiments (Fig. 3*C*). After consulting the authors of Gauthier-Coles *et al.* (6) and considering reports by Jakobsen *et al.* (7) of potential equilibrium solubility issues with MMTC/57E above 2 μM, we also performed a subsequent experiment by preserial diluting the MMTC/57E stock in dimethyl sulfoxide (DMSO) to reach a final assay concentration of 20 μM and a final DMSO concentration of 5% in all conditions. Although in earlier experiments, turbidity was noted briefly upon the addition of the concentrated stock that resolved within a few seconds of mixing end to end, under these prediluted conditions, no turbidity was noticed. Assay controls (+SNAT2, KO, and saturating alanine) were not affected by the inclusion of 5% DMSO, and in agreement with our aforementioned data, MMTC/57E still failed to inhibit SLC38A2-dependent MeAIB uptake in our established HY15549 iSNAT2 cell line (Fig. 3*C*, *experiment indicated as filled black circles*).

### Characterizing SNAT2 deficiency using unbiased metabolomics

**Figure 4:**
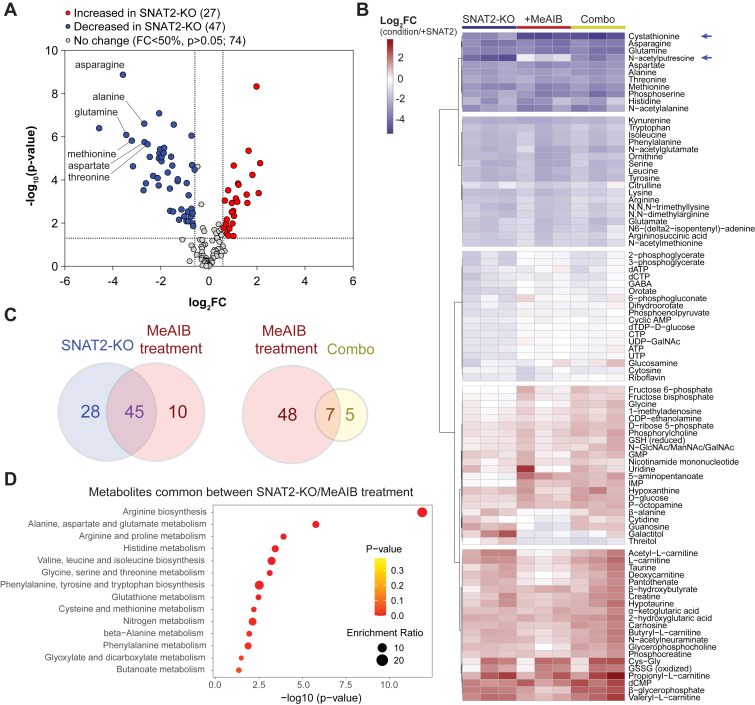
**SNAT2 deficiency because of knockout or inhibition by MeAIB causes broad changes in amino acid metabolism.***A*, Volcano plot of metabolomic changes in HY15549 iSNAT2 cells cultured with or without doxycycline for 24 h. Neutral and other amino acids represent the most dramatically decreased metabolites and, in some cases, are indicated. Metabolite changes were considered significant if the absolute log_2_ fold change (FC) was >1.5, *p* < 0.05, and false discovery rate (FDR) <10%. *B*, heatmap depicting log_2_ FC in metabolite abundances in SNAT2-KO, MeAIB, or combination of HY15549 iSNAT2 cells relative to + SNAT2 (+doxycycline, 0.5 μg/ml). Only metabolites that passed a significance threshold (*p* < 0.05 and FDR <10%) in at least one condition were included in the heatmap. *Blue arrows* indicate metabolites (cystathionine, *N*1-acetylputrescine) that were MeAIB or SNAT2-KO dominant. *C*, Venn diagram comparing the significantly different metabolites between SNAT2-KO and MeAIB treatment (*left*) or MeAIB-treated + SNAT2 or SNAT2-KO (Combo) (*right*). *D*, metabolites that were commonly affected by SNAT2-KO or MeAIB treatment were subjected to a metabolite set enrichment analysis using 80 curated metabolite sets based on the Kyoto Encyclopedia of Genes and Genomes (KEGG) human metabolic pathways (MetaboAnalyst, accessed December 2024). Data in Figure 2 are mean ± SD of n = 3 independent experiments and n = 2 biological replicate wells. MeAIB, α-(methylamino)-isobutyric acid; SNAT2, sodium-coupled neutral amino acid transporter 2.

Unbiased metabolomics using LC–MS is a powerful tool to characterize metabolic responses to pharmacological, nutrient, genetic, or environmental stressors. We sought to establish a metabolomic signature associated with SNAT2 deficiency, which may be used to evaluate on-target and potential off-target engagement of pharmacological SNAT2 inhibitors. To this end, we maintained HY15549 iSNAT2 cells in 0.5 μg/ml dox (+SNAT2) or without dox (SNAT2-KO) prior to quenching, metabolite extraction, and analysis. Of the 148 detected metabolites, 74 were significantly altered with an absolute fold change greater than 1.5 following acute SNAT2 withdrawal (*p* < 0.05, false discovery rate <0.1) (Fig. 4*A*). Consistent with our previously reported GC–MS-based amino acid analysis (5), proteinogenic amino acids and direct downstream metabolic intermediates were among the most significantly decreased in SNAT2-KO cells including SNAT2 substrates and nonsubstrates (*e.g.*, aspartate) (Fig. 4*A*). Other significantly reduced metabolites include nucleotides, nucleoside phosphates, glutathione metabolites, and glycolytic intermediates (Fig. 4*B*). To determine if the competitive inhibition of SNAT2 results in a similar metabolic response to SNAT2-KO, we treated HY15549 iSNAT2 cells (+dox) with 10 mM of MeAIB for 18 h. We identified 55 metabolites that were significantly altered using a similar significance cutoff as before; valine was removed from further analysis because of its chromatographic coelution with isomeric MeAIB (Fig. 4*B*). There was a significant overlap in the metabolic alterations caused by SNAT2-KO (45/73) and MeAIB treatment (45/55), including a significant decrease in many amino acids (Fig. 4*C*). Enrichment analysis using the Kyoto Encyclopedia of Genes and Genomes human metabolic pathways set revealed that the 45 commonly affected metabolites were enriched in amino acid metabolic pathways (Fig. 4*D*). Interestingly, *N*-acetyl-putrescine, a polyamine derivative, was significantly decreased in SNAT2-KO cells but less affected by MeAIB treatment (Fig. 4*B*). Polyamines are reported as potential cancer biomarkers, including pancreatic (17) and lung (18) cancers, and acetylated polyamines also influence free acetyl-CoA pools and protein acetylation (19). Notably, glutathione metabolites were significantly enriched in the SNAT2-KO and MeAIB-treated samples, including reduced and oxidized glutathione and the degradation intermediate Cys-Gly dipeptide (Fig. 4, *B* and *D*). Other antioxidants, including taurine and hypotaurine (20), as well as several mitochondrial acyl-carnitine species were also significantly increased in SNAT2-KO cells relative to controls (Fig. 4*B*). We also treated SNAT2-KO cells with 10 mM MeAIB (“Combo”), as before, to assess the relative selectivity of MeAIB for SNAT2. Consistent with our earlier results that identified SNAT2 as the exclusive system A transporter expressed in this isogenic cell model, only 12 metabolites were significantly affected, with 7/12 representing an MeAIB-specific effect (Fig. 4, *B* and *C*). Notably, MeAIB treatment in either condition caused a dominant decrease in cystathionine levels, which may be attributed to an SNAT2-independent effect (Fig. 4*B*). These results provide insights into the metabolic effects of SNAT2 on amino acid metabolism and describe a specific metabolite signature associated with SNAT2 inhibition.

### MMTC/57E-treated cells do not exhibit an SNAT2-deficient metabolite profile

**Figure 5:**
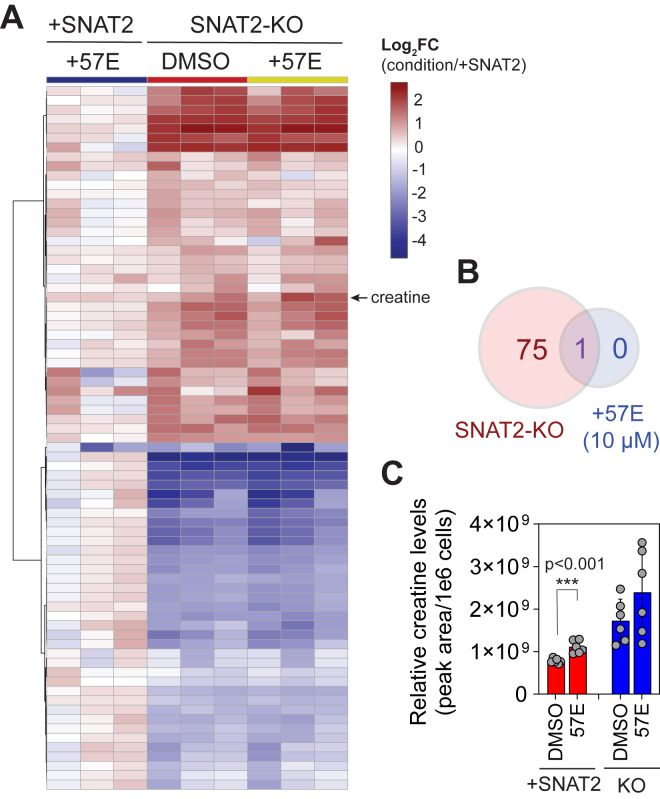
**MMTC/57E-treated cells do not exhibit a metabolic profile consistent with on-target inhibition of SNAT2.***A*, heatmap depicting log_2_ fold change in metabolite abundances in MMTC/57E-treated (10 μM) + SNAT2 cells or SNAT2-KO cells treated with vehicle (DMSO) or MMTC/57E (10 μM) relative to vehicle-treated + SNAT2 (+doxycycline, 0.5 μg/ml) cells. Only metabolites that passed a significance threshold (*p* < 0.05 and FDR <10%) in at least one condition were included in the heatmap. Only one metabolite (creatine) was significantly affected by MMTC/57E treatment, which is indicated with a *black arrow* and *label*. *B*, Venn diagram depicting the number and overlap of metabolites that were significantly affected by SNAT2-KO or MMTC/57E treatment. *C*, relative creatine abundance, normalized by the number of cells analyzed. Data in (*A* and *C*) are mean ± SD of n = 3 independent experiments. DMSO, dimethyl sulfoxide; FDR, false discovery rate; MMTC/57E, 3-(*N*-methyl(4-methylphenyl)sulfonamido)-*N*-(2-trifluoromethylbenzyl)thiophene-2-carboxamide; SNAT2, sodium-coupled neutral amino acid transporter 2.

To assess whether MMTC/57E-treated cells exhibit a similar metabolomic signature to SNAT2-deficient cells, we cultured HY15549 iSNAT2 cells with or without dox for 6 h followed by treatment with either 10 μM MMTC/57E or vehicle (DMSO) for 18 h. Although vehicle-treated SNAT2-KO cells exhibited a similar metabolic defect as observed earlier, only a single metabolite (creatine) was significantly increased in MMTC/57E-treated, SNAT2-expressing cells using a similar significance cutoff as before (Fig. 5, *A–C*). These data, together with those derived from monitoring MeAIB uptake, do not support that MMTC/57E is an SNAT2 inhibitor.

### Validation of the MMTC/57E identity and solubility using LC–MS

**Figure 6:**
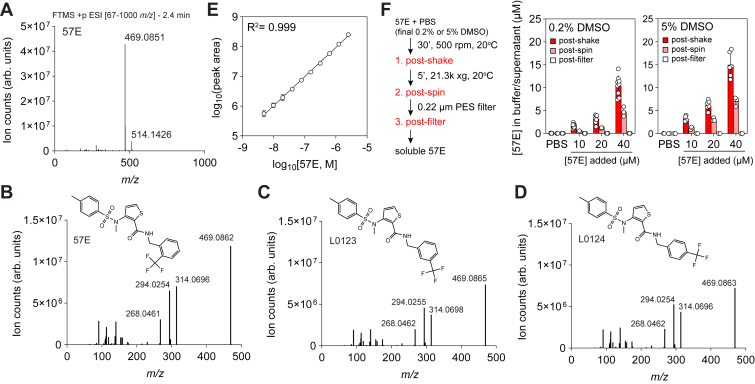
**LC–MS verification of the identity of aqueous solutions of MMTC/57E and isomers.***A*, representative MS1 spectrum of a peak with room temperature of 2.4 min corresponding to the expected *m/z* for MMTC/57E in positive mode. *B*–*D*, representative MS2 fragmentation spectra of parent ion 469.0862 *m/z* at ∼2.4 min for MMTC/57E (*D*), L0123 (*E*), and L0124 (*F*) showing similar fragmentation patterns. The structures for each isomer are overlayed. *E*, standard curve to determine the MMTC/57E concentration using LC–MS (*R*^2^ = 0.999). A MMTC/57E stock at 2.5 μM was prepared in LC mobile phase (80% acetonitrile) and serially diluted to ∼4.9 nM. *F*, solubility analysis of MMTC/57E in PBS. Samples of MMTC/57E were prepared at 10, 20, or 40 μM in PBS at a final DMSO concentration of 0.2% or 5% and collected after vortexing in a polystyrene tissue culture dish, after centrifugation, and after filtration using a 0.2 μm polyethersulfone (PES) filter; all steps were performed at room temperature (20 °C). The concentration of MMTC/57E in the supernatant of each sample was quantified using the standard curve in (*E*). Data in (*A*–*D*) are representative from n = 3 independent experiments. Data in (*E*) are mean ± SD of n = 2 independently prepared serial dilutions. Data in (*F*) are mean ± SD of n ≥ 1 independent experiments and n = 3 biological replicates. DMSO, dimethyl sulfoxide; MMTC/57E, 3-(*N*-methyl(4-methylphenyl)sulfonamido)-*N*-(2-trifluoromethylbenzyl)thiophene-2-carboxamide.

Confirming the identity of small molecules, particularly when negative results are observed, is critical to determine if small molecules degrade or suffer from batch inconsistencies. Therefore, we analyzed an aliquot of our transport assay media using high-resolution tandem MS following an MeAIB uptake experiment. We also included 2 *n*-substituted trifluoromethylbenzyl isomer of MMTC/57E (L0123, L0124) to compare *m/z* and fragmentation patterns. We identified peaks in both positive and negative modes at a retention time of ∼2.5 min that matched the expected *m/z* of the proton adduct of MMTC/57E. The peak in positive mode was more abundant with an [M + H] = 469.0862 *m/z* ion, which corresponds to a mass error of ∼1.1 ppm (expected 469.0867 *m/z*) within the mass accuracy of our LC–MS (Fig. 6*A*). Analysis of L0123 and L0124 isomers resulted in similar retention time and *m/z*. Fragmentation of the parent ion peaks for MMTC/57E, L0123, and L0124 results in rich, highly similar fragmentation spectra and peak abundances across the 3 isomers (Fig. 6, *B–D*). These results provide confirmation of the identity of MMTC/57E used in our assays.

Jakobsen *et al.* (7) reported that MMTC/57E exhibited poor equilibrium solubility (<1.8 μM) and significant precipitation upon addition of DMSO stocks to aqueous buffers. We performed a similar solubility experiment whereby MMTC/57E stocks were prepared in PBS at 10, 20, or 40 μM with a final DMSO concentration of either 0.2% or 5%. A standard curve was prepared by transferring MMTC/57E directly from the 20 mM DMSO stock to a glass vial containing LC–MS mobile phase (80% acetonitrile in water). The resulting standard curve resulted in a linear response to the lowest assayed concentration of 4.9 nM (Fig. 6*E*). Stocks of MMTC/57E in PBS were added to polystyrene tissue culture plates used in earlier studies, and we collected samples following an incubation at room temperature for 30 min, after centrifugation, and after filtering using a 0.2 μm hydrophilic, polyethersulfone syringe filter (Fig. 6*F*). Each sample was added directly to 80% acetonitrile and analyzed by LC–MS. The concentration of MMTC/57E quantified in PBS after shaking using the standard curve was significantly less than what was added for all 3 concentrations tested, indicating significant binding of MMTC/57E to plastic (Fig. 6*F*). Notably, brief centrifugation to remove large particles decreased the concentration of MMTC/57E quantified in the supernatant (Fig. 6*F*). No MMTC/57E was detected after filtering, suggesting significant precipitation (Fig. 6*F*). Our results are consistent with those reported in the study by Jakobsen *et al.* and confirm that MMTC/57E exhibits poor aqueous solubility.

## Discussion

Amino acid transporters have underdeveloped pharmacology compared with other transmembrane protein families (*e.g.*, G-protein coupled receptors, ion channels). This is in part because of a lack of reliable assays with which to interrogate large chemical libraries to identify potent and selective inhibitors and to characterize target engagement. However, several solute carriers (SLCs) that fuel cell metabolism have associated clinical pharmacological inhibitors that are used to treat a variety of medical conditions, such as diabetes (SLC5A2/SGLT2), major depressive and other psychological conditions (selective serotonin reuptake inhibitors; SLC6A4/hSERT), and hypertension and edema (SLC12A1/NKC22; SLC12A3/NCC) (21). Although recent developments in high-throughput assay technologies allow the interrogation of large chemical libraries on a specific transporter, the redundancy in substrate transport by SLCs poses a challenge for studying SLC-specific uptake and identifying specific analog inhibitors. For instance, in U87-MG glioma and A549 cells, alanine uptake is facilitated by SNAT1, SNAT2, SNAT5, SLC6A15 (B0AT2), SLC7A6 (y+LAT2), SLC1A4 (ASCT1), and SLC1A5 (ASCT2), and many of these transporters are also involved in glutamine transport (22). Several tool inhibitors of amino acid transporters exist, but they often mimic endogenous substrates and have relatively low affinity (*e.g.*, MeAIB).

In this study, we describe 2 cell-based assays to monitor SNAT2 activity, which can be used to assess inhibitor target engagement. One of the major challenges in identifying transporter inhibitors is the potential redundancy of amino acid transporters that are coexpressed in any given cell line used for screening and/or hit confirmation. The expression of other system A transporters, like SNAT1, may interfere with the identification of a specific SNAT2-deficient metabolite signature or inhibitors that are selective in high-throughput screens. We have validated that the HY15549 iSNAT2 cell line lacks endogenous SNAT1 expression and likely other transporters that are inhibited by MeAIB (*e.g.*, SNAT4). This exclusive expression profile may be due to the procedure used to generate the cell line, as we attempted to minimize selective pressures and maintain an isogenic background. While the characterization of endogenous SNAT4 expression and activity is challenging because of a lack of validated reagents, our results strongly support that the uptake of MeAIB and its metabolic impacts in this context are primarily dependent upon SNAT2 and provide a selective readout of its activity. Other approaches to profile putative SNAT2 inhibitor responses in nonisogenic cell lines suffer risks associated with adaptive changes in redundant transporter expression profiles.

Using both cell-based SNAT2 activity assays, we showed that MMTC/57E fails to elicit a response that is consistent with its report as an SNAT2 inhibitor (6). The poor solubility of MMTC/57E is one possible explanation for our negative results but highlights that MMTC/57E is not a useful small-molecule tool to dissect the importance of SNAT2 for cancer cell metabolism and growth using conventional approaches. Future studies may be needed to investigate formulations using encapsulation agents (*e.g.*, cyclodextrin), which improve aqueous solubility to confirm MMTC/57E as a tool inhibitor of SNAT2 (23). Other possible explanations could be the presence of impurities in screening stocks that exhibit inhibitory activity, as was observed during the identification of inhibitors that selectively target the renal outer medullary potassium channel (24). In this case, resynthesis and reconfirmation are necessary during the hit confirmation stage for a drug discovery campaign. Although there are no other independently validated SNAT2 inhibitors reported, robust assays, including those reported herein, may enable the discovery of potent and selective inhibitors in the future.

## Experimental procedures

### Cell culture

Cell cultures were maintained in Dulbecco's modified Eagle's medium (DMEM) supplemented with 10% fetal bovine serum (FBS) and 1% penicillin–streptomycin (pen–step) in a 5% CO_2_ humidified incubator and passaged every 2 to 3 days using 0.25% trypsin–EDTA at an approximate 1:10 split ratio. For LC–MS metabolomics experiments, a homemade dialyzed FBS was used, which was generated using 3.5 kDa molecular weight cutoff dialysis tubing and four, 12- to 24-h incubations at 4 °C with ultrapure water, including a final incubation in 0.9% NaCl; each dialysis was performed at 20× v/v. The parental HY15549 cell line was previously isolated from female KPC tumors (25). The isogenic HY15549 iSNAT2 cell line was derived by plating single cells in 96-well plates and subcloned in DMEM containing dox (0.5 μg/ml), which was refreshed every 5 days, to express SNAT2 during clonal selection as previously described (5). Briefly, 6 individual clones were pooled after individual validation for KO and dox-inducible SNAT2 expression using Western blot. HY15549 iSNAT2 and 293T cell lines were generously provided by Dr Alec Kimmelman (NYU School of Medicine). All cell cultures were routinely verified to be negative for *Mycoplasma* by PCR and prior to freezing of cell stocks.

### Molecular cloning and lentiviral transduction

To clone the lentiviral expression vector for human SNAT1, we used Gateway LR Clonase II (Invitrogen; catalog no.: 11791020) to combine pDONR221-SLC38A1-STOP (Addgene; catalog no.: 161261) and pLenti CMV Blast DEST (Addgene; catalog no.: 17451) and transformed homemade competent Stbl3 *Escherichia coli* (Invitrogen; catalog no.: C737303). Competent cells were homemade using the Mix & Go! *E. coli* Transformation kit (Zymo Research; catalog no.: T3001). Lentivirus was produced by transfecting 293T cells with +SNAT1 or EV pLenti CMV Blast DEST, pMD2.G (Addgene; catalog no.: 12259), and psPAX2 (Addgene; catalog no.: 12260) using Opti-MEM I (Thermo Fisher Scientific; catalog no.: 31985070) and a standard Lipofectamine 3000 (Thermo Fisher Scientific; L3000008) transfection protocol. After an overnight transfection, the transfection media were removed from 293T cells and replaced with DMEM supplemented with 10% FBS and 1% pen–strep. After 48 h, the viral supernatant was harvested, filtered using a 0.45 μm syringe filter, aliquoted, and stored at −80 °C. To transduce HY15549 iSNAT2 cells with either EV or +SNAT1 lentivirus, cells were treated with 10 μg/ml hexadimethrine bromide (Sigma; catalog no.: 107689) and an aliquot of lentiviral supernatant. Following an overnight transduction, cells were selected with 10 μg/ml Blasticidin S (Sigma; catalog no.: 15205) until nontransduced controls were fully eliminated, approximately 7 days.

### Antibodies and Western blot

Proteins were extracted using radioimmunoprecipiattion assay buffer (Sigma; catalog no.: 20-188) containing fresh protease cocktail (Roche; catalog no.: 11836170001) using a refrigerated vortexer for approximately 30 min. Crude lysates were cleared by centrifugation at 4 °C, aliquoted, and stored at −80 °C before analysis by SDS-PAGE. The protein concentration in each lysate was quantified using a DC Protein Assay (Bio-Rad; catalog no.: 5000112) against a concentration gradient of bovine serum albumin (Sigma; catalog no.: A7030). Lysates were prepared for SDS-PAGE in Tris–Glycine SDS sample buffer (Thermo Fisher Scientific; LC2676) and not boiled, as heating above 50 °C causes complete loss of SNAT2 signal (5). Protein transfer was confirmed by staining with Ponceau S and imaged using an iBright FL1500 imager (Thermo Fisher Scientific) after brief rinsing with distilled water. Membranes were then blocked in 5% nonfat milk (Medallion Milk Company) prepared in Tris-buffered saline with Tween according to the antibody manufacturer’s recommendations. Primary antibodies were incubated overnight at 4 °C with gentle agitation using the following antibodies and dilutions: anti-SNAT1 (1:1000 dilution, Cell Signaling Technologies; catalog no.: 36057), anti-SNAT2 (1:1000 dilution, MBL; catalog no.: BMP081), anti-N/K-ATPase (1:10,000 dilution, Abcam; catalog no.: ab76020), or anti-Actin (1:10,000 dilution, Sigma; catalog no.: A4700). After washing with Tris-buffered saline with Tween, membranes were incubated with peroxidase-conjugated secondary antibodies, anti-rabbit (1:5000 dilution, Cell Signaling Technologies; catalog no.: 7074) or anti-mouse (1:20,000 dilution, Cell Signaling Technologies; catalog no.: 7076) for 1 h at room temperature and imaged by chemiluminescence (Thermo Fisher Scientific; catalog no.: 34577) using an iBright FL1500 imager.

### MMTC/57E solubility analysis using LC–MS

MMTC/57E (L876–0122), L0123 (L876–0123), and L0124 (L876–0124) were acquired from ChemDiv and prepared as 20 mM stocks in DMSO (Sigma; catalog no.: D2650). Stocks were aliquoted and stored in −80 °C. For all studies, aliquots were thawed by bath sonication for 4 min at room temperature and freeze/thawed a maximum of 3 times before disposal. Stocks of 5, 10, and 20 mM MMTC/57E were prepared fresh for each solubility experiment using DMSO. Each stock (2 μl) was added to 1 ml of PBS (for 0.2% final DMSO) or PBS containing 5% DMSO (for 5% final DMSO), mixed, and added to a polystyrene, tissue culture–treated 12-well plates (Greiner; catalog no.: 665180). Each plate was vortexed on a plate shaker at ∼500 rpm for 30 min at 20 °C. After 30 min, 10 μl of the postshake sample was added to 90 μl of 80% HPLC-grade acetonitrile and 20% HPLC-grade water. The remaining volume was transferred to a 1.5 ml tube and centrifuged for 5 min at 21,300*g* at 20 °C. The postspin sample (10 μl) was added to 90 μl of 80% HPLC-grade acetonitrile and 20% HPLC-grade water. The postspin supernatant (500 μl) was transferred to a 1 ml syringe with a 0.2 μm, 13 mm diameter polyethersulfone filter (Cytiva Acrodisc Supor; catalog no.: 4602) attached. The postspin sample was filtered into a new 1.5 ml tube, and 10 μl of the postfilter sample was added to 90 μl of 80% HPLC-grade acetonitrile and 20% HPLC-grade water. All samples were prepared in glass vial inserts for analysis by LC–MS.

### MeAIB uptake using GC–MS

HY15549 iSNAT2 cells were plated onto 6-well plates at 3e5 cells/well with or without dox (0.5 μg/ml) and incubated for 24 h before starting the uptake assay. Plates were brought to room temperature for 15 min before washing twice with room temperature in PBS and adding 2 ml of transport assay buffer, consisting of PBS containing a concentration gradient of MeAIB. For inhibition studies using l-alanine or MMTC/57E, cells were pretreated with 1 ml of PBS and 0.5 ml of PBS containing l-alanine, MMTC/57E, or DMSO for 15 min at room temperature before adding 0.5 ml of PBS containing MeAIB (0.5 mM). For the studies to quantify the IC_50_ of l-alanine, cells were cotreated with l-alanine from 0 to 20 mM and 0.5 mM MeAIB for 30 min at room temperature before washing (3×; 0.9% NaCl), metabolite extraction, and analysis. All sodium-free uptake assays were performed by replacing sodium with an equimolar amount of *N*-methyl-d-glucamine (Sigma; catalog no.: 66930) in PBS, and pH was adjusted using KOH and KCl. All assays were performed at a physiological pH of 7.2 to 7.4. Once MeAIB was added, plates were incubated for 30 min at room temperature and quenched by washing twice with room temperature in PBS and adding 1 ml of ice-cold 80% HPLC-grade methanol in HPLC-grade water containing 0.4 μl of stable isotope–labeled amino acid standards (Cambridge Isotope Labs; catalog no.: MSK-A2), 1 nmol of ^13^C_5_,^15^N_2_-glutamine (Cambridge Isotope Labs; catalog no.: CNLM-1275-H), 1 nmol of ^13^C_4_,^15^N_2_-asparagine:H2O (Cambridge Isotope Labs; catalog no.: CNLM-3819-H), and 1 nmol of 2,4,5,6,7-^2^H_5_-tryptophan (CDN Isotopes; catalog no.: D-1522) per well. For l-alanine IC_50_ experiments, metabolites were extracted by adding 1 ml of ice-cold 80% HPLC-grade methanol in HPLC-grade water containing 2.5 nmol of ^13^C_3_-l-alanine (Cambridge Isotope Labs; catalog no.: CLM-2184-H-PK). Cells were scraped using a p1000 tip, and metabolite extracts were transferred to a tube, vortexed for approximately 10 to 15 min at 4 °C, and centrifuged using a refrigerated centrifuge at 4 °C. The cleared metabolite extracts (925 μl) were transferred to a new tube and concentrated using a SpeedVac (Thermo Fisher; catalog no.: SPD120) until dry for analysis by GC–MS.

To measure MeAIB uptake using GC–MS, we first derivatized metabolites into their methoxime ester TBDMS derivatives by adding 25 μl of methoxyamine hydrochloride (20 mg/ml, in pyridine; prepared fresh) and incubating at 37 °C for 1 h followed by a 5-min incubation at room temperature with 25 μl of MTBSTFA + 1% TBDMS chloride (Sigma; catalog no.: 375934). Derivatives were centrifuged for 15 min to remove insolubles, and 40 μl was transferred to a sample vial for analysis. GC–MS analysis was performed using an Agilent 8890 gas chromatograph with a DB-35MS column (30 m × 0.25 mm i.d. × 0.25 μm; Agilent; catalog no.: 122-3832UI) interfaced with an inert, deactivated fused silica MSD transfer column (5 m × 0.15 mm i.d.; Agilent, catalog no.: CP801505). The GC temperature was adjusted to the following parameters: 75 °C held after injection, ramped to 255 °C at 7.5 °C, held at 255 °C or 10 min, ramped to 320 °C at 10 °C/min, and held for 5 min. The MS was operated in full-scan mode from 100 to 650 *m/z*. Peak heights corresponding to MeAIB (174 *m/z*, 13.6 min) and isotopically labeled internal standards were extracted and quantified using previously published algorithms (26). Intracellular MeAIB was normalized to the ^13^C_3_,^15^N-labeled alanine internal standard signal.

### Metabolomics

HY15549 iSNAT2 cells were plated onto 6-well plates at 3e5 cells/well in DMEM containing 10% of dialyzed FBS and 1% pen–strep supplemented with or without dox (0.5 μg/ml). Cells were allowed to attach for approximately 6 h in a humidified CO_2_ incubator at 37 °C. After 6 h, the media were completely aspirated and replaced with assay media containing final concentrations of MeAIB, MMTC/57E, or DMSO (as a vehicle for MMTC/57E experiments) and incubated for 18 h. To quench and extract metabolites from cell cultures, media were aspirated, and cells were washed twice with ice-cold 0.9% NaCl in HPLC-grade water. After complete aspiration, 1 ml of ice-cold 80% methanol containing 0.4 μl of stable isotope–labeled amino acid standards (Cambridge Isotope Labs; catalog no.: MSK-A2), 1 nmol of ^13^C_5_,^15^N_2_-glutamine (Cambridge Isotope Labs; catalog no.: CNLM-1275-H), 1 nmol of ^13^C_4_,^15^N_2_-asparagine:H2O (Cambridge Isotope Labs; catalog no.: CNLM-3819-H), and 1 nmol of 2,4,5,6,7-^2^H_5_-tryptophan (CDN Isotopes; catalog no.: D-1522) was added to each well. Plates were processed individually and briefly stored at −80 °C until all assay plates were quenched. Cell metabolites were harvested and dried using a similar approach as the GC–MS experiments. Once dry, metabolites were reconstituted into 50 μl of HPLC-grade water and vortexed at 4 °C for 15 min before centrifugation at 4 °C for 15 min. The supernatant (40 μl) was transferred to glass sample vial inserts (Agilent; catalog no.: 5183-2088), avoiding any insoluble pellets, for LC–MS analysis.

### LC–MS analysis

Sample vials were loaded into a temperature-controlled autosampler at 6 °C and subjected to LC–MS analysis. A SeQuant ZIC-pHILIC (150 × 2.1 mm; Sigma; catalog no.: 1504600001) LC column was coupled to a Thermo Scientific Vanquish HPLC. Some analyses were performed with a SeQuant ZIC-pHILIC guard column (20 × 2.1 mm; Sigma; catalog no.: 1504380001) installed in front of the analytical column. The volume of oven temperature was set to 25 °C, maintained by forced air and an integrated column heater. The column was pre-equilibrated using a flow rate of 100 μl/min and 80% to 20% B. Following injection of 2 μl of sample, the following gradient elution at 100 μl/min was used: 80% to 20% B (0–30 min), 20% to 20% B (30–40 min), and 20% to 80% B (40–40.5 min); the LC column was re-equilibrated using 80% to 80% B from 40.5 to 52 min before subsequent injections. Mobile phase composition was (A) 10 mM ammonium carbonate in HPLC-grade water, pH 9.0 and (B) acetonitrile, 100%. Mobile phase A was freshly prepared or used within 1 week.

The LC was coupled to a Thermo Scientific Exploris 240 mass spectrometer operating in heated electrospray ionization mode for analysis. The following parameters were set for heated electrospray ionization mode: spray voltage 3.4 kV (positive) and 2 kV (negative), static spray voltage, sheath gas 25, auxiliary gas 5, sweep gas 0.5, ion transfer tube temperature 320 °C, and vaporizer temperature 75 °C. The global parameters included an expected peak width of 20 s, mild trapping, and a default charge state of 1. A 40-min polarity switching data-dependent Top 5 method was used for positive mode and a data-dependent Top 3 method was used for negative mode. Full MS scan parameters for both positive and negative modes were set as follows: scan range of 67 to 1000 *m/z* collected in profile mode, Orbitrap resolution 120,000, RF lens 70%, automatic gain control (AGC) target of 300%, and maximum injection time set to automatic. ddMS2 for positive mode were collected in centroid mode at an Orbitrap resolution of 30,000, isolation window of 1.5 *m/z*, an AGC target set to standard, a maximum injection time set to automatic, and a normalized collision energy set to 10%, 30%, and 80%. ddMS2 for negative mode were collected in centroid mode at an Orbitrap resolution of 30,000, isolation window of 2 *m/z*, an AGC target set to standard, a maximum injection time set to automatic, and a normalized collision energy set to 30%. For both positive and negative ddMS2, we applied an intensity threshold of 5e4 and a dynamic exclusion of 5 ppm for 10 s, excluding isotopes.

Raw LC–MS data were analyzed using TraceFinder (Thermo Fisher Scientific). Peaks corresponding to specific metabolites were annotated using a retention time and accurate mass library created from the Mass Spectrometry Metabolite Library of Standards (IROA Technologies), and other authentic standards acquired from Sigma. Peak areas were used to quantify the relative abundance of each metabolite, unless specified as peak height, and were quantified using the following general ICIS algorithm settings: an *m/z* discrimination threshold of 5 ppm, a retention time window of 60 or 120 s, a minimum peak area of 5e5, a smoothing factor of 9, an area noise factor of 5, a peak noise factor of 10, a baseline window of 100, a minimum peak height signal-to-noise ratio of 2, a minimum peak width of 5, a multiplet resolution of 10, an area tail extension of 20, and an area scan window of 0. Each peak integration was manually inspected, and individual settings were adjusted to optimize integration. Peak areas/heights were normalized by cell number following each treatment, which was quantified from a surrogate-treated plate after processing metabolites for analysis.

### Statistical analysis

The statistical test performed for each comparison and the type of replication are stated in each corresponding figure legend. No technical sampling was performed. Independent experiments were considered an experiment performed on a separate day on separately plated cells. *p* Values and/or the representative star annotations are listed in each figure and figure legend. Statistical analysis of LC–MS metabolomics analysis was performed using MetaboAnalyst (https://www.metaboanalyst.ca/), and filtering parameters are listed in the text and figure legends. Other statistical analyses were performed using Prism (version 10.3.1; GraphPad Software, Inc).

## Data availability

Raw LC–MS and GC–MS data files are available at the National Metabolomics Data Repository as study IDs (ST003996, ST004013, ST004014, ST004016, ST004018, and ST004022) and are publicly available as of the date of publication. The GC–MS analysis script is available at https://github.com/Sethjparker/IntegrateNetCDF_WithCorrect (accessed February 6, 2025) under Massachusetts Institute of Technology license. Any additional information required to reanalyze the data reported in this article is available from the corresponding author (seth.parker@bcchr.ca) upon request.

## Conflict of interest

S. J. P. is involved in the discovery of pharmacological inhibitors targeting SNAT2. While the results presented in this article are negative for reported SNAT2 inhibitors, we declare that there is no direct financial conflict of interest related to the findings of this study.
